# Supplementary material for: Content-rich biological network constructed by mining PubMed abstracts
Source: BMC Bioinformatics. 2004 Oct 8;5:147. doi: 10.1186/1471-2105-5-147 (PMC528731; doi:10.1186/1471-2105-5-147)
Supplement: Additional File 2 — The original results of the above study (non-essential files are deleted to keep the file size under the limit set by BMC bioinformatics). [file 1471-2105-5-147-S2.bz2 › chilibotAdditionalFile2/dip05/675ID9261125E11081/html/BMPR1B_BMP7.html]

 


 **BMPR1B** and **BMP7** 
  
Found 10 abstracts in PubMed, retrieved 05.  
 

 What does Google say? 
 PDF only 
| .edu only 

---

**Interactive relationship** (e.g. stimulation, inhibition, etc)

**Neutral relationship**- We therefore propose that the effects of OP 1  [ **BMP7** ]  on these cells in vitro are mediated by ALK 6  [ **BMPR1B** ]  BMPR IB.  Ref: 10705576 Growth Factors, 2000

**Non-interactive relationship** (e.g. studied together, co-existance, homology, etc.)

- Correlation between ALK 6  [ **BMPR1B** ]  BMPR IB distribution and responsiveness to osteogenic protein 1  [ **BMP7** ]  BMP 7  [ **BMP7** ]  in embryonic mouse bone rudiments.  Ref: 10705576 Growth Factors, 2000
- The temporary high immunostaining for ALK 6  [ **BMPR1B** ]  in the early proliferating chondrocytes and future perichondrium of E14 bone rudiments, and its absence in older bones correlated with the sensitivity of chondrocytes and perichondrium to exogenous OP 1  [ **BMP7** ] .  Ref: 10705576 Growth Factors, 2000
- We assessed the distribution and relative immunohistochemical staining intensity of the bone morphogenetic protein 7  [ **BMP7** ] , osteogenic protein 1  [ **BMP7** ]  OP 1  [ **BMP7** ] , in its pro and mature forms, and four of its receptors, type I ALK 2, ALK 3, and ALK 6  [ **BMPR1B** ]  and type II in normal adolescent New Zealand White rabbit articular cartilage.  Ref: 12364567 J Histochem Cytochem, 2002
- We then investigated whether the sensitivity of the early long bone rudiments to OP 1  [ **BMP7** ]  correlated with high expression of the OP 1  [ **BMP7** ]  binding type I serine threonine kinase receptors activin receptor like kinase ALK 2 ActR I, ALK 3 BMPR IA or ALK 6  [ **BMPR1B** ]  BMPR IB at this early stage.  Ref: 10705576 Growth Factors, 2000
